# Altitude-Linked Distribution Patterns of Serum and Hair Mineral Elements in Healthy Yak Calves from Ganzi Prefecture

**DOI:** 10.3390/vetsci12080718

**Published:** 2025-07-31

**Authors:** Chenglong Xia, Yao Pan, Jianping Wu, Dengzhu Luorong, Qingting Yu, Zhicai Zuo, Yue Xie, Xiaoping Ma, Lan Lan, Hongrui Guo

**Affiliations:** 1 College of Veterinary Medicine, Sichuan Agricultural University, Wenjiang, Chengdu 611130, China; xiachenglong@stu.sicau.edu.cn (C.X.); czyhzzc@126.com (Z.Z.); xyue@sicau.edu.cn (Y.X.); mxp886@sicau.edu.cn (X.M.); 2 Animal Husbandry Science Institute of Ganzi Tibetan Autonomous Prefecture, Kangding 626000, China; chenzirui1027@163.com (Y.P.); xukesuo303@163.com (J.W.); lrdz@163.com (D.L.); Yuqingtingxukesuo@163.com (Q.Y.)

**Keywords:** yak calves, hair, serum, trace elements, altitude, Ganzi Prefecture

## Abstract

Mineral imbalances in livestock can critically impair growth, immunity, and productivity. Yaks inhabiting the Qinghai–Tibetan Plateau face unique environmental challenges, including high-altitude-induced nutrient variability. This study investigated the status of mineral elements and their correlations with altitude in healthy yak calves across five regions in Ganzi Prefecture, located at elevations ranging from 3100 to 4100 m. Hair and serum samples from 35 calves were analyzed for 11 essential elements (Na, K, Ca, Mg, S, Cu, Fe, Mn, Zn, Co, and Se). The results revealed widespread deficiencies. Key deficiencies were identified: hair Na and Co were significantly below references value (*p* < 0.05), and Se was consistently deficient across all regions, with deficiency rates ranging from 35.73% to 56.57%. Serum Mg and Cu were generally deficient (Mg deficiency > 26% above 3800 m). S, Mn (low detection), and Co were also suboptimal. Serum selenium deficiency was notably severe in lower-altitude areas (≤59.07%). Significant correlations with altitude were observed: hair sodium levels decreased with increasing altitude (r = −0.72), while hair manganese (r = 0.88) and cobalt (r = 0.65) levels increased. Serum magnesium deficiency became more pronounced at higher elevations (r = 0.58), whereas selenium deficiency in serum was more severe at lower altitudes (r = −0.61). These findings indicate prevalent multi-element deficiencies in yak calves that are closely linked to altitude and are potentially influenced by soil mineral composition and feeding practices, as suggested by previous studies. The study underscores the urgent need for region-specific nutritional standards and altitude-adapted mineral supplementation strategies to support optimal yak health and development.

## Simple Summary

Yaks are essential livestock in the Qinghai–Tibet Plateau, especially in Ganzi Prefecture, where they provide meat, milk, and economic security for local herders. However, the high-altitude environment presents unique nutritional challenges, particularly concerning mineral element deficiencies. This study examined the levels of 11 important macro- and trace elements in the hair and serum of healthy yak calves from five regions with different altitudes (3100–4100 m). We found that deficiencies in elements such as selenium, cobalt, magnesium, and sodium were common, and some were closely related to altitude. Hair and serum samples offered complementary insights—serum reflected short-term status, while hair showed long-term mineral balance. These results highlight the need for targeted mineral supplementation based on altitude to improve yak health and productivity. Our findings offer practical guidance for yak farmers and veterinary professionals working in highland environments.

## 1. Introduction

The yak (*Bos grunniens*), a species uniquely adapted to the Qinghai–Tibet Plateau and adjacent high-altitude regions, serves as a cornerstone of animal husbandry in Ganzi Prefecture, Sichuan Province [1,2]. Renowned for its physiological resilience, the yak thrives in harsh environments characterized by hypoxia, low temperatures, and intense ultraviolet radiation. However, this adaptation relies heavily on the internal homeostasis of various essential mineral elements. Deficiencies in these elements can impair physiological function and productivity, ultimately leading to substantial economic losses for herders [3]. Due to the extensive, grazing-based husbandry system predominant in these plateau regions, ensuring the adequate intake of essential nutrients remains challenging [4,5]. In addition, seasonal fluctuations in forage availability, regional soil mineral content, and altitude-related ecological variability contribute to inconsistent patterns of nutritional deficiency among different herding areas [6]. Accurate nutritional assessment requires localized sampling and targeted analysis to inform region-specific supplementation strategies. To date, limited research has systematically evaluated mineral element status specifically in yak calves across altitude gradients, leaving a critical knowledge gap regarding juvenile mineral homeostasis in highland environments. This study addresses this gap by providing foundational data to guide targeted nutritional interventions.

Serum analysis is commonly used to assess the mineral status of livestock, as it reflects the current physiological state and enables the dynamic monitoring of nutrition or disease responses. Although less frequently employed, hair analysis offers complementary value by reflecting long-term mineral deposition and metabolic trends [7]. Hair is easy to collect, stable under storage, and suitable for retrospective mineral evaluation. Certain elements, such as zinc and selenium, accumulate in higher concentrations in hair, making variations more detectable [8]. Furthermore, studies suggest correlations between mineral levels in hair and serum for select elements [9]. Thus, the combined use of serum and hair analysis allows a more comprehensive assessment of both short-term dynamics and long-term mineral status, particularly in the context of environmental influences such as altitude gradients [10].

Maintaining mineral homeostasis—both in macroelements and trace elements—is essential for the health and productivity of livestock [11]. These elements are widely distributed in animal tissues and play critical roles in physiological structure and metabolic function. Routine monitoring of their concentrations is vital. However, recent yak-related mineral studies have predominantly focused on adult animals or disease-related contexts. Systematic investigations in yak calves, a critical stage of growth and development, remain limited. Moreover, few studies have quantitatively evaluated how altitude influences mineral status. Reference values for elements such as cobalt and selenium are not standardized, and detection guidelines for some (e.g., serum cobalt) are ambiguous, complicating the formulation of evidence-based nutritional interventions. Regarding altitude–mineral relationships, conflicting findings exist: while some studies report that high altitudes exacerbate deficiencies due to reduced mineral content in forage, others suggest that specific deficiencies may be more pronounced at lower altitudes due to soil composition or anthropogenic effects.

This study aims to investigate the mineral element status in healthy yak calves from five distinct highland areas in Ganzi Prefecture—Yajiang, Daofu, Litang, Luhuo, and Jiulong (altitude range: 3100–4100 m). By analyzing 11 key mineral elements in hair and serum samples, we characterize deficiency patterns and spatial distribution across altitudinal gradients. The findings provide scientific evidence to guide targeted mineral supplementation and ecological management strategies, supporting yak health and promoting sustainable livestock practices in high-altitude pastoral systems.

## 2. Materials and Methods

### 2.1. Experimental Animals

This study was conducted in five high-altitude pastoral regions of Ganzi Prefecture, Sichuan Province, China. The selected locations and their respective altitudes were as follows: Yajiang county (3600 m), Daofu county (3100 m), Litang county (3860 m), Luhuo county (3200 m), and Jiulong county (4100 m). These regions represent a typical altitudinal gradient within the yak-grazing plateau ecosystem.

A total of 35 clinically healthy yaks aged between 1 and 2 years (locally referred to as “calves,” as animals under 2 years are generally categorized as juvenile or immature in local herding practices) were randomly selected, 7 from each study site. All animals were evaluated by local veterinary staff and exhibited no visible signs of disease or malnutrition at the time of sampling.

### 2.2. Hair Samples Collection

Hair sample collection procedures followed standard veterinary methods as described by Sizova [7] and Lim [8], ensuring consistency with established sampling protocols. Approximately 10 g of hair was collected from the dorsal neck region of each animal using clean stainless steel scissors. To avoid external contamination, the sampling area was cleaned prior to collection. The samples were sealed in sterile polyethylene bags and stored at −20 °C until analysis.

### 2.3. Serum Samples Collection

Serum sample collection procedures followed standard veterinary methods as described by Sizova [7] and Lim [8], ensuring consistency with established sampling protocols. Jugular blood was collected aseptically using vacuum tubes without anticoagulant. Samples were transported on ice and centrifuged at 3000 rpm for 10 min within 6 h of collection. The separated serum was aliquoted and stored at −80 °C until mineral analysis.

### 2.4. Mineral Element Analysis

Each hair and serum sample was homogenized, and an appropriate amount was weighed into a microwave digestion vessel. Subsequently, 6 mL of analytical-grade nitric acid (HNO_3_) was added, and samples were left to stand overnight for pre-digestion. Digestion was performed using the microwave digestion system. After cooling, digested samples were transferred into volumetric flasks and diluted to 25 mL with ultrapure water. An Inductively Coupled Plasma Optical Emission Spectrometer (ICP-OES) was used for the determination of the following: sodium (Na), potassium (K), calcium (Ca), magnesium (Mg), iron (Fe), manganese (Mn), zinc (Zn), and sulfur (S). An Inductively Coupled Plasma Mass Spectrometer (ICP-MS) was used for the detection of the following: cobalt (Co) and selenium (Se).

All elemental standard solutions used in this study were obtained from the National Nonferrous Metals and Electronic Materials Analysis and Testing Center, each with a certified concentration of 1000 μg/mL (k = 2). These included:

Potassium (K): GSB-04-1733-2004

Calcium (Ca): GSB-04-1720-2004

Sodium (Na): GSB-04-1738-2004

Magnesium (Mg): GSB-04-1735-2004

Iron (Fe): GSB-04-1726-2004

Manganese (Mn): GSB-04-1736-2004

Copper (Cu): GSB-04-1725-2004

Zinc (Zn): GSB-04-1761-2004

Selenium (Se): GSB-04-1751-2004

Cobalt (Co): GSB-04-1722-2004

Sulphur: GBW(E)085593

Reference values for mineral concentrations were primarily adopted from the published veterinary literature on yaks. For elements lacking established reference values (e.g., sodium in hair), the average measured value from healthy adult yaks in comparable environmental settings was used as a reference benchmark.

### 2.5. Data Analysis

All data were processed using Microsoft Excel and SPSS Statistics 26.0. Descriptive statistics (mean ± standard deviation) were calculated for each element. A Pearson correlation analysis was used to examine the relationships between mineral concentrations and altitude.

The deficiency rate (%) for each element was calculated using the following formula:

Deficiency Rate (%) = (Reference Value − Measured Value)/Reference Value × 100%

## 3. Results

### 3.1. Hair Macroelement Profiles in Yaks Across an Altitudinal Gradient

Data on the macroelement content in yak hair from the five Ganzi regions are shown in Table 1. Reference values were cited from the literature [2,3] or the average content of normal yaks. Reference values for Na, K, Ca, Mg, and S in hair were 682.51, 66.54, 1989, 130, and 30,000 mg/kg, respectively. The data show that the sodium content in Yajiang and Luhuo was significantly higher than the reference value (846.1456 and 994.4428 mg/kg). Deficiencies of varying degrees existed in Daofu, Litang, and Jiulong. Jiulong had the most severe hair sodium deficiency (deficiency rate 47.5%), followed by Litang (25.3%), while Daofu was in a suboptimal state (deficiency rate 9.4%). Calcium was not deficient in any region, with Luhuo significantly higher (3931.5432 mg/kg) than the other four. Magnesium content was above the reference value in all five regions, showing no deficiency. Sulfur was also above the reference value in all regions, indicating no deficiency.

The correlation analysis between the four macroelement contents in yak hair and altitude is shown in Table 2. Hair sodium content showed a negative correlation with altitude (r = −0.73, *p* < 0.01), decreasing as altitude increased.

### 3.2. Analysis of Trace Element Content in Yak Hair and Correlation with Altitude

Data on trace element content in yak hair from the five Ganzi regions are shown in Table 3. Reference values were cited from the literature [9,12]: Cu, Fe, Mn, Zn, Co, and Se were 6.42, 489, 13, 100, 0.98, and 0.52 mg/kg, respectively. The data show that the average hair Cu, Fe, and Mn content in all five regions were above reference values, indicating no deficiency. Zinc levels in Daofu, Jiulong, and Luhuo (98.4810, 101.6163, and 99.1166 mg/kg) were slightly below the reference value (102.37 mg/kg), with deficiency rates of 3.8%, 0.8%, and 3.2%, respectively, indicating suboptimal status. Average cobalt content in Yajiang, Daofu, Litang, Luhuo, and Jiulong was 0.1963, 0.2317, 0.4197, 0.257, and 0.3197 mg/kg, with deficiency rates of 74.17%, 69.51%, 44.78%, 66.18%, and 57.93%, respectively, indicating deficiency in all regions. Selenium was deficient in all five regions, with average contents of 0.3149, 0.2128, 0.2893, 0.2509, and 0.2777 mg/kg and deficiency rates of 35.73%, 56.57%, 40.96%, 48.8%, and 43.33%, respectively.

The correlation analysis between the six trace element contents in yak hair and altitude is shown in Table 4. Manganese and cobalt content in hair correlated with altitude. Manganese was not deficient, but its content increased with increasing altitude (r = 0.88, *p* < 0.01). The cobalt deficiency status correlated with altitude (r = 0.65, *p* < 0.05), worsening at lower altitudes.

### 3.3. Analysis of Serum Macroelement Content in Yaks and Correlation with Altitude

Data on five serum macroelement contents in yaks from the five Ganzi regions are shown in Table 5. Reference values were cited from the literature [12,13,14,15]: Na, K, Ca, Mg, and S were 3000–3500, 90–237, 90, 25, and 1276 mg/L, respectively. Potassium and calcium were above reference values in all five regions, showing no deficiency. Sodium in Jiulong averaged 2975.51 mg/L, slightly below the lower reference limit (3000 mg/L), with a deficiency rate of 0.8%. Magnesium levels in the five regions were 23.9073, 22.8389, 19.8703, 18.4574, and 19.323 mg/L, with deficiency rates of 4.4%, 8.6%, 20.5%, 26.2%, and 22.7%, respectively. This indicates suboptimal status in Yajiang and Daofu and deficiency in Litang, Luhuo, and Jiulong. Sulfur showed varying degrees of deficiency in all regions, with average contents of 1013.7869, 1064.9791, 1109.035, 1119.7598, and 1153.6591 mg/L and deficiency rates of 20.5%, 16.5%, 13.1%, 12.2%, and 9.6%, respectively. Jiulong was suboptimal, while the other four regions showed deficiency.

Correlation analysis between the five serum macroelement contents and altitude is shown in Table 6. Only magnesium content correlated significantly with altitude (r = 0.58, *p* < 0.05), with deficiency worsening as altitude increased.

### 3.4. Analysis of Serum Trace Element Content in Yaks and Correlation with Altitude

Serum trace element content data in yaks are shown in Table 7. Reference values for Cu, Fe, Mn, Zn, Co, and Se in serum were 0.6–1.2, 1.1, 0.006, 0.6, 0.4, and 0.07 mg/L, respectively [3,4,5,6]. Among the six trace elements measured, Zn and Fe levels were above reference values in all regions. Iron in Jiulong was notably high (4.0249 mg/L), significantly exceeding other regions, suggesting potential iron excess. Copper was deficient in all five regions, with deficiency rates of 45.8%, 44.8%, 64.3%, 47.0%, and 41.2%, respectively. Manganese deficiency was more severe; only 3 out of 35 samples had detectable levels, the rest were below the instrument’s detection limit. Cobalt deficiency existed in all five regions to varying degrees, with deficiency rates of 20.7%, 28.9%, 30.8%, 80.8%, and 49.9%. Luhuo had significantly higher deficiency than other regions, followed by Jiulong, Litang, Daofu, and Luhuo. Serum selenium in Luhuo was above the reference value (normal level). Deficiency rates in Yajiang, Daofu, Litang, and Jiulong were 57.7%, 78.9%, 48.8%, and 66.5%, respectively.

As shown in Table 8, among the six serum trace elements measured, only selenium content correlated significantly with altitude (r = −0.61, *p* < 0.05), with deficiency increasing as altitude decreased.

## 4. Discussion

This study identified varying degrees of zinc, cobalt, and selenium deficiencies in yak hair across five regions. In serum, deficiencies were more extensive, involving sodium, magnesium, sulfur, copper, manganese, cobalt, and selenium. These findings underscore the importance of region-specific mineral supplementation strategies tailored to localized deficiency profiles to safeguard yak calf health and development.

Previous studies have shown that sodium, a vital electrolyte, is involved in numerous physiological functions including fluid balance and cellular activity. Deficiency can result in lethargy, reduced appetite, poor coat condition, and in severe cases, neurological symptoms [16]. Our findings suggest that the hair analysis revealed progressive sodium deficiency in Daofu, Litang, and Jiulong, indicative of chronic underconsumption. Serum sodium levels were marginally low only in Jiulong, suggesting a more acute insufficiency, whereas Daofu and Litang maintained serum sodium within reference ranges, potentially due to recent dietary adjustments.

Magnesium is a cofactor for many enzymes involved in various physiological and biochemical reactions and plays important roles in cardiovascular protection and bone health [17]. Magnesium deficiency can cause irritability, tremors, frothing, and convulsions in cattle, affecting their health. Our findings suggest that hair test results showed magnesium content above reference values in all five regions, within normal levels. However, serum tests revealed varying degrees of deficiency in all regions: Yajiang and Daofu were slightly below reference (suboptimal), while Litang, Luhuo, and Jiulong were significantly deficient.

Sulfur is an essential component of sulfur-containing amino acids involved in protein structure, the synthesis of coenzymes and vitamins, and the formation of connective tissue [18]. Our findings suggest that the hair test results showed normal sulfur levels in all regions. Serum test results revealed sulfur deficiency in all regions. We recommend that appropriate sulfur supplementation be implemented based on the detected levels in each region.

Copper is a key component of the active centers of many enzymes, widely involved in energy metabolism, antioxidant functions, and the production of elastin and collagen [19]. Copper deficiency can easily lead to cardiovascular disease, reduced immune response in cattle, growth retardation and diarrhea in calves, reduced fertility and sperm quality in bulls, and impaired steroid hormone synthesis in ovarian granulosa cells [20,21,22]. Our findings suggest that hair copper content was normal in all five regions, but serum copper was severely deficient in all regions.

Manganese is crucial for bone and cartilage development and participates in antioxidant functions, reproduction, and metabolism [23]. Manganese deficiency affects estrus in cattle, reduces conception rates, decreases birth weight, and increases abortion risk [24]. Low manganese can also lead to paralysis and bone damage [20]. Our findings suggest that hair manganese content was normal, but serum manganese was extremely deficient: only 3 out of 35 samples across the five regions had detectable data; concentrations in the rest were below the instrument’s detection sensitivity. We recommend that manganese intake be supplemented as a priority.

Zinc, as the active center of many enzymes, participates in metabolic activities and plays a significant role in immune regulation. Clinical manifestations of zinc deficiency mainly occur in calves, including weak hooves, interdigital dermatitis or foot rot, reduced conception rates, severe impairment of sperm maturation, reduced feed intake and growth rate, lethargy, decreased immunity, and parakeratosis [25]. In our study, we found that serum zinc content was within normal levels in all five regions, whereas the hair zinc levels in Daofu, Jiulong, and Luhuo were slightly below reference, indicating a suboptimal status. We suggest that daily feeding requires attention to a balanced nutritional intake.

Previous studies have shown that cobalt is the core element of vitamin B12, which participates in red blood cell maturation, thereby affecting the body’s hematopoietic function. It is also involved in energy metabolism, protein synthesis, and other functions. Cobalt deficiency can cause loss of appetite, rough and dull hair, anemia, poor development, and emaciation. In our study, we found that both hair and serum test results showed severe cobalt deficiency in all five regions, highlighting the need for cobalt supplementation.

The main physiological function of selenium is as the core component of glutathione peroxidase (GPX), catalyzing the decomposition of peroxides and acting synergistically with vitamin E as an antioxidant, playing a vital role in protecting cell structure and protein synthesis [26]. Previous studies have shown that selenium deficiency primarily affects the normal growth and development of calves, hinders the metabolism and utilization of fat and vitamin E, and mainly causes myocardial and skeletal muscle necrosis, with affected muscle areas losing their original color and appearing pale (white muscle disease) [27]. Our findings suggest that selenium levels were severely deficient in both hair and serum across all five regions. We emphasize that long-term selenium deficiency poses a serious threat to calf health, and selenium supplementation should be highly prioritized.

Our findings indicate that the hair sodium negatively correlated with elevation, indicating that higher-altitude yaks are more sodium deficient. Additionally, we observed that hair manganese and cobalt positively correlated with altitude, suggesting greater deficiencies at lower elevations. Furthermore, our study showed that serum magnesium positively correlated with altitude, pointing to a worsening magnesium deficiency at higher elevations. Finally, we found that serum selenium showed a significant negative correlation with altitude, suggesting that low-altitude yaks are more prone to selenium deficiency.

This study has several limitations. The relatively small sample size (35 animals) and geographic restriction to Ganzi Prefecture may limit the generalizability of the findings. Moreover, the absence of direct forage and soil mineral analyses constrains our understanding of environmental contributions. Future research should address these gaps by incorporating larger sample sizes and comprehensive environmental assessments.

## 5. Conclusions

In conclusion, this study aimed to evaluate the altitude-associated distribution patterns of key mineral elements in yak calves from Ganzi Prefecture, with the goal of identifying nutritional deficiencies and informing targeted supplementation strategies. Our findings revealed that deficiencies in sodium, magnesium, cobalt, selenium, copper, and manganese were widespread, with notable variations linked to altitude. These insights highlight the urgent need for region-specific and altitude-adapted nutritional interventions to support the health and productivity of yak calves in high-altitude pastoral systems. Future research should expand sample sizes and incorporate environmental assessments to further refine nutritional recommendations.

## Figures and Tables

**Table 1:** Macroelement content (mg/kg, mean ± SD) in yak hair samples across five regions compared to reference values.

Element	Reference	Yajiang (3600 m)	Daofu (3100 m)	Litang (3860 m)	Luhuo (3200 m)	Jiulong (4100 m)
Na (mg/kg)	682.51	846.145 ± 483.897	618.597 ± 150.787	509.821 ± 351.899	994.442 ± 568.182	358.431 ± 181.625
Ca (mg/kg)	1989	2898.128 ± 772.237	2529.115 ± 651.117	2683.063 ± 244.212	3931.543 ± 378.869	2641.380 ± 620.558
Mg (mg/kg)	285	611.553 ± 196.997	523.132 ± 128.902	541.741 ± 70.468	640.690 ± 58.332	656.848 ± 126.105
S (mg/kg)	30,000	34,027.5037 ± 3988.225	34,595.379 ± 3807.227	32,480.217 ± 1199.769	34,790.772 ± 1145.851	38,083.197 ± 1347.465

**Table 2:** Correlation coefficients (r) and *p*-values between yak hair macroelement concentrations (mg/kg, mean ± SD) and altitude across five regions.

Element	Correlation Coefficient (r)	*p*-Value	Significant	Correlation Interpretation
Na	−0.72	<0.01	**	Significant negative correlation; content decreases with increasing altitude
Ca	0.34	0.42	Not Significant	No significant correlation
Mg	0.22	0.61	Not Significant	No significant correlation
S	0.09	0.85	Not Significant	No significant correlation

**Table 3:** Trace element content (mg/kg, mean ± SD) in yak hair samples across five regions compared to reference values.

Element	Reference	Yajiang (3600 m)	Daofu (3100 m)	Litang (3860 m)	Luhuo (3200 m)	Jiulong (4100 m)
Cu (mg/kg)	6.42	8.112 ± 0.722	8.828 ± 3.022	6.785 ± 1.221	8.444 ± 0.491	6.366 ± 0.894
Fe (mg/kg)	391	480.094 ± 289.462	638.191 ± 694.780	910.489 ± 453.448	493.213 ± 124.284	806.195 ± 535.782
Mn (mg/kg)	15.93	42.298 ± 30.037	33.503 ± 18.190	85.666 ± 15.514	59.253 ± 22.034	35.339 ± 17.552
Zn (mg/kg)	102.37	124.162 ± 14.858	98.481 ± 9.220	115.410 ± 15.073	101.616 ± 6.155	99.116 ± 6.876
Co (mg/kg)	0.76	0.196 ± 0.117	0.231 ± 0.204	0.419 ± 0.109	0.257 ± 0.054	0.319 ± 0.179
Se (mg/kg)	0.49	0.314 ± 0.088	0.212 ± 0.074	0.289 ± 0.059	0.251 ± 0.020	0.277 ± 0.088

**Table 4:** Correlation coefficients (r) and *p*-values between yak hair trace element concentrations (mg/kg, mean ± SD) and altitude across five regions.

Element	Correlation Coefficient (r)	*p*-Value	Significant	Correlation Interpretation
Cu	−0.41	0.34	Not Significant	No significant correlation
Fe	0.15	0.75	Not Significant	No significant correlation
Mn	0.88	<0.01	**	Significant positive correlation; content increases with increasing altitude
Zn	−0.27	0.53	Not Significant	No significant correlation
Co	0.65	<0.05	*	Significant positive correlation; deficiency worsens at lower altitude
Se	0.12	0.81	Not Significant	No significant correlation

**Table 5:** Serum macroelement concentrations (mg/L, mean ± SD) in yak calves across five regions compared to reference values.

**Element**	**Reference**	**Yajiang (3600 m)**	**Daofu (3100 m)**	**Litang (3860 m)**	**Luhuo (3200 m)**	**Jiulong (4100 m)**
Na (mg/L)	3000–3500	3139.329 ± 63.265	3144.662 ± 97.363	3115.320 ± 151.299	3106.203 ± 147.933	2975.515 ± 552.671
K (mg/L)	97–234	171.855 ± 15.550	159.863 ± 16.997	177.505 ± 14.084	167.943 ± 25.899	148.195 ± 16.594
Ca (mg/L)	90	100.982 ± 6.290	108.256 ± 10.811	100.240 ± 16.660	100.089 ± 8.471	95.475 ± 11.472
Mg (mg/L)	25	23.907 ± 3.435	22.838 ± 3.088	19.870 ± 1.890	18.457 ± 1.411	19.323 ± 1.851
S (mg/L)	1276	1013.786 ± 116.929	1064.979 ± 117.599	1109.035 ± 76.689	1119.759 ± 53.807	1153.659 ± 55.375

**Table 6:** Correlation coefficients (r) and *p*-values between yak serum macroelement concentrations (mg/L, mean ± SD) and altitude across five regions.

Element	Correlation Coefficient (r)	*p*-Value	Significant	Correlation Interpretation
Na	−0.08	0.89	Not Significant	No significant correlation
K	0.14	0.78	Not Significant	No significant correlation
Ca	−0.21	0.63	Not Significant	No significant correlation
Mg	0.58	<0.05	*	Significant positive correlation; deficiency worsens with increasing altitude
S	0.50	0.18	Not Significant	No significant correlation

**Table 7:** Serum trace element concentrations (mg/L or μg/L, mean ± SD) in yak calves across five regions compared to reference values.

Element	Reference	Yajiang (3600 m)	Daofu (3100 m)	Litang (3860 m)	Luhuo (3200 m)	Jiulong (4100 m)
Cu (mg/L)	0.6–1.2	0.325 ± 0.100	0.331 ± 0.059	0.214 ± 0.116	0.317 ± 0.080	0.352 ± 0.110
Fe (mg/L)	1.1	1.432 ± 0.490	2.649 ± 1.512	1.318 ± 0.524	2.539 ± 1.4387	4.024 ± 1.462
Mn (mg/L)	0.006	0.006 ± 0.016	0	0	0	0.007 ± 0.012
Zn (mg/L)	0.6	1.096 ± 0.126	1.677 ± 0.402	1.448 ± 0.325	1.195 ± 0.264	1.359 ± 0.549
Co (μg/L)	0.4	0.317 ± 0.332	0.284 ± 0.145	0.276 ± 0.204	0.076 ± 0.063	0.200 ± 0.131
Se (mg/L)	0.07	0.029 ± 0.018	0.0147 ± 0.004	0.035 ± 0.005	0.158 ± 0.003	0.023 ± 0.005

**Table 8:** Correlation coefficients (r) and *p*-values between yak serum trace element concentrations (mg/L or μg/L, mean ± SD) and altitude across five regions.

Element	Correlation Coefficient (r)	*p*-Value	Significant	Correlation Interpretation
Cu	−0.23	0.62	Not Significant	No significant correlation
Fe	0.45	0.26	Not Significant	No significant correlation
Mn	−0.15	0.75	Not Significant	No significant correlation
Zn	0.37	0.38	Not Significant	No significant correlation
Co	0.193	0.34	Not Significant	No significant correlation
Se	−0.61	<0.05	*	Significant negative correlation; deficiency worsens with decreasing altitude

## Data Availability

The data that support the findings of this study are available from the corresponding author upon reasonable request.

## References

[1] Guo X., Long R., Kreuzer M., Ding L., Shang Z., Zhang Y., Yang Y., Cui G. (2014). Importance of functional ingredients in yak milk-derived food on health of Tibetan nomads living under high-altitude stress: A review. Crit. Rev. Food Sci..

[2] Shah A.M., Bano I., Qazi I.H., Matra M., Wanapat M. (2023). “The Yak”—A remarkable animal living in a harsh environment: An overview of its feeding, growth, production performance, and contribution to food security. Front. Vet. Sci..

[3] Lou Y., Yang T., Zhu Y., Xia C., Cui H., Deng H., Huang Y., Fang J., Zuo Z., Guo H. (2024). Effects of Trace Elements and Vitamins on the Synthesis of Steroid Hormones in Follicular Granulosa Cells of Yak. Vet. Sci..

[4] Lean I.J., Golder H.M. (2023). Pasture Minerals for Dairy Cattle. Vet. Clin. N. Am. Food Anim. Pract..

[5] Sapkota S., Acharya K.P., Laven R., Acharya N. (2022). Possible Consequences of Climate Change on Survival, Productivity and Reproductive Performance, and Welfare of Himalayan Yak (*Bos grunniens*). Vet. Sci..

[6] Donghai F. (2020). Analysis of Seasonal Variations and Surplus and Shortage of Partial Trace Elements in the Soil-Forage-Yak System in Gannan Region. China Herbiv. Sci..

[7] Sizova E.A., Miroshnikov S.A., Notova S.V., Marshinskaya O.V., Kazakova T.V., Tinkov A.A., Skalny A.V. (2022). Serum and Hair Trace Element and Mineral Levels in Dairy Cows in Relation to Daily Milk Yield. Biol. Trace Elem. Res..

[8] Lim H.J., Lee S., Park W., Park E., Yoo J.G. (2024). Mineral patterns in hair: A decisive factor between reproducible and repeat breeder dairy cows. PLoS ONE.

[9] Fang X. (2020). Effects of Rib and Age on Serum and Hair Mineral Contents and Correlation of Mineral Contents between Serum and Hair of Jinchuan Yak. Chin. J. Anim. Nutr..

[10] Grace N.D., Knowles S.O. (2012). Trace element supplementation of livestock in new zealand: Meeting the challenges of free-range grazing systems. Vet. Med. Int..

[11] Brugger D., Windisch W.M. (2015). Environmental responsibilities of livestock feeding using trace mineral supplements. Anim. Nutr..

[12] Suocheng W. (2007). Determination of Mineral Elements and Blood Biochemical Parameters in Gannan Yak. J. Northwest Minzu Univ. (Nat. Sci. Ed.).

[13] Fan Q., Wanapat M., Hou F. (2019). Mineral Nutritional Status of Yaks (*Bos grunniens*) Grazing on the Qinghai-Tibetan Plateau. Animals.

[14] Xin G.S. (2010). The Mineral Dynamics of Soil-Plant-Animal System from Northeast of the Qinghai-Tibetan Plateau.

[15] Han Z., Li R., Li K., Shahzad M., Wang X.Q., Jiang W., Luo H., Qiu G., Nabi F., Li J. (2016). Assessment of Serum Trace Elements in Diarrheic Yaks (*Bos grunniens*) in Hongyuan, China. Biol. Trace Elem. Res..

[16] Whitlock R.H., Kessler M.J., Tasker J.B. (1975). Salt (sodium) deficiency in dairy cattle: Polyuria and polydipsia as prominent clinical features. Cornell Vet.

[17] Dalgliesh A.J., Liu Z.Z., Griffiths L.G. (2017). Magnesium Presence Prevents Removal of Antigenic Nuclear-Associated Proteins from Bovine Pericardium for Heart Valve Engineering. Tissue Eng. Part A.

[18] Hill C.R., Shafaei A., Balmer L., Lewis J.R., Hodgson J.M., Millar A.H., Blekkenhorst L.C. (2023). Sulfur compounds: From plants to humans and their role in chronic disease prevention. Crit. Rev. Food Sci..

[19] Nair P.M., Srivastava R., Mani V., Arulkumar S., Tyagi N., Mondal G. (2025). The importance of zinc, copper and manganese and their impact on growth, immunity and fertility of male cattle: A review. Biometals.

[20] Xue Y. (2016). The Effect of Copper, Manganese and Iodine on Yak’s Rumen Fermentation, Blood Indexes and Growth Performance.

[21] Clauss M., Dierenfeld E.S. (1999). Susceptibility of yak (*Bos grunniens*) to copper deficiency. Vet. Rec..

[22] Lou Y., Yang T., Xia C., Yang S., Deng H., Zhu Y., Fang J., Zuo Z., Guo H. (2025). Effects of Copper on Steroid Hormone Secretion, Steroidogenic Enzyme Expression, and Transcriptomic Profiles in Yak Ovarian Granulosa Cells. Vet. Sci..

[23] Nair P.M., Srivastava R., Chaudhary P., Kuraichya P., Dhaigude V., Naliyapara H.B., Mondal G., Mani V. (2023). Impact of zinc, copper, manganese and chromium supplementation on growth performance and blood metabolic profile of Sahiwal (*Bos indicus*) male calves. Biometals.

[24] Rojas M.A., Dyer I.A., Cassatt W.A. (1965). Manganese Deficiency in the Bovine. J. Anim. Sci..

[25] Li W. (2016). Effects of Iron, Zinc and Selenium on Rumen Fermentation, Growth Performance and Blood Biochemical Parameters of Yak. Master’s Thesis.

[26] Mehdi Y., Dufrasne I. (2016). Selenium in Cattle: A Review. Molecules.

[27] Rederstorff M., Krol A., Lescure A. (2006). Understanding the importance of selenium and selenoproteins in muscle function. Cell Mol. Life Sci..

